# GBDTCDA: Predicting circRNA-disease Associations Based on Gradient Boosting Decision Tree with Multiple Biological Data Fusion

**DOI:** 10.7150/ijbs.33806

**Published:** 2019-11-08

**Authors:** Xiujuan Lei, Zengqiang Fang

**Affiliations:** School of Computer Science, Shaanxi Normal University, Xi'an 710119, China

**Keywords:** circRNA-disease associations, machine learning, Gradient Boosting, multiple biological data

## Abstract

Circular RNA (circRNA) is a closed-loop structural non-coding RNA molecule which plays a significant role during the gene regulation processes. There are many previous studies shown that circRNAs can be regarded as the sponges of miRNAs. Thus, circRNA is also a key point for disease diagnosing, treating and inferring. However, traditional experimental approaches to verify the associations between the circRNA and disease are time-consuming and money-consuming. There are few computational models to predict potential circRNA-disease associations, which become our motivation to propose a new computational model. In this study, we propose a machine learning based computational model named Gradient Boosting Decision Tree with multiple biological data to predict circRNA-disease associations (GBDTCDA). The known circRNA-disease associations' data are downloaded from cricR2Disease database (http://bioinfo.snnu.edu.cn/CircR2Disease/). The feature vector of each circRNA-disease association pair is composed of four parts, which are the statistics information of different biological networks, the graph theory information of different biological networks, circRNA-disease associations' network information and circRNA nucleotide sequence information, respectively. Therefore, we use those feature vectors to train the gradient boosting decision tree regression model. Then, the leave one out cross validation (LOOCV) is adopted to evaluate the performance of our computational model. As for predicting some common diseases related circRNAs, our method GBDTCDA also obtains the better results. The Area under the ROC Curve (AUC) values of Basal cell carcinoma, Non-small cell lung cancer and cervical cancer are 95.8%, 88.3% and 93.5%, respectively. For further illustrating the performance of GBDTCDA, a case study of breast cancer is also supplemented in this study. Thus, our proposed method GBDTCDA is a powerful tool to predict potential circRNA-disease associations based on experimental results and analyses.

## Introduction

Circular RNAs (circRNAs) are one kind of short non-coding RNAs 1 which have no exposed terminating 5'-cap and 3'-polyadenylated tail structures and are closed loops, which are unlike the linear RNAs that have terminated with 5′ caps and 3′ tails 2. It is this closed loops structure that makes more difficult to detect circRNAs in organisms 3-5. At the same moment, the closed loops structure makes circRNA more stable and conversed to be regarded as a biomarker to mark some diseases. With the development of basic sequence technologies and high-throughput technologies, more and more circRNAs functions are revealed 6. Many studies have shown that circRNAs can work as the sponges for competing endogenous RNAs or miRNAs 7-9, which makes circRNAs also can be treated as diseases biomarkers. Secondly, circRNAs also have effects on the alternative splicing and transcription process by isolating the translation start position to modulate protein expression 10, 11. Thirdly, circRNAs are involved in modulating the expression of parental genes 12. What's more, circRNAs also function as the retinol-binding protein (RBP) sponges, which can strengthen the interaction between the MBL protein and circMbl 10 or get involve in translating as templates. In addition, increasing numbers of evidences and studies have shown that circRNAs play significant roles in disease diagnosis and treatment 13. Especially for various cancers 14, 15, cardiovascular disease 16, diabetes 17, etc. Current researches of the associations between circRNAs and diseases are relatively advanced works, which might be based on the pathway circRNA-miRNA-mRNA to explore potential knowledge.

Recently, people pay more and more attention to exploring complicated associations between circRNAs and other biological molecules such as circRNA-miRNA, circRNA-lncRNA, and so on. In order to better promote the researches of circRNA, some useful databases are constructed to collect the information of circRNAs, which include circBase (http://www.circbase.org/) 18, Circ2Traits (http://gyanxet-beta.com/circdb/) 19 and circR2Disease (http://bioinfo.snnu.edu.cn/CircR2Disease/) 20. CircRNA can obtain highly nuclease-resistant ability because of the particular closed loops structures. A stable closed loop structure of circRNAs also helps circRNAs own longer half-lives than the usual linear RNAs 21, which can be regarded as a unique property to diagnose some circRNA-related diseases. Although traditional RNA-Seq techniques are used widely to detect diseases related circRNAs and high-throughput techniques are adopted to validate, which can help us obtain some accurate experimental results, these techniques are still expensive and time-consuming. There are few computational models to detect potential or promising circRNA-disease associations simultaneously, which is also our motivation to develop this study.

In this study, we adopt the gradient boosting decision tree 22 to predict potential circRNA-disease associations, which is named Gradient Boosting Decision Tree with multiple biological data to predict circRNA-disease associations (GBDTCDA). Multiple biological data such as circRNAs related expression profile data, gene ontology (GO) terms data and base sequence are adopted to construct circRNA similarity network (*CSN*). Diseases related ontology terms and genes are involved in building disease similarity network (*DSN*). Then the statistics information of *CSN*, *DSN* and circRNA-disease associations network, the graph theory information of *CSN* and *DSN*, representational biological indicators of circRNA, such as GC content and K-mer, other information like latent vectors extracted from cicrRNA-disease association network are regarded as feature vector to indicate each circRNA-disease pair. Some of those feature vectors are input to train the model and the rest of data are treated as test data. Here, leave one out cross validation (LOOCV) is adopted to evaluate the performance of GBDTCDA. The area under ROC curve (AUC) value of LOOCV is 0.834, which is a better result than other machine learning methods or network-based methods. In order to further illustrate the performance of GBDTCDA, we also make some case studies. Therefore, GBDTCDA is a powerful method to predict the potential circRNA-disease associations.

## Materials & Methods

### Human circRNA-disease associations

In this study, human disease-related circRNAs are extracted from the initial dataset which is downloaded from CircR2Disease database 23 (http://bioinfo.snnu.edu.cn/CircR2Disease/). All the collected circRNA-disease associations are validated by biological experiments in CircR2Disease database. There are 739 circRNA-disease associations are collected in the database where includes 661 circRNA entries and 100 disease entries, which are composed of the initial dataset. Then, we pick up the distinct 140 cicrRNA-disease associations involving 132 circRNAs and 40 diseases, which could be regarded as a suitable known numbers of circRNA-disease associations in the circRNA-disease associations' matrix. Thus, matrix *A* is utilized to describe the circRNA-disease associations. If there is an existing association between the circRNA *c*(*i*) and disease *d*(*j*),* A*(*c*(*i*), *d*(*j*)) is equal to 1, otherwise it is equal to 0. The related data used is shown in table 1.

### CircRNA similarity

#### CircRNA sequence similarity

To calculate circRNA sequence similarity, the circRNA related sequence data is downloaded from circBase database 18. There are 132 circRNA sequence data picked up from circBase database based on the circRNA ID of the circR2Disease database. After we get the circRNA sequence data, a sequence alignment algorithm called the Needleman-Wunsch pairwise alignment algorithm is used to calculate the circRNA sequence similarity. Needleman-Wunsch pairwise algorithm is integrated into a python package called Biopython 24. The parameter gap-open penalty and gap-open extending penalty are set as 2, -0.5 to -0.1 respectively. Then we describe the matrix *Seq_CS* as the circRNA sequence similarity matrix.

#### CircRNA functional annotation semantic similarity

Gene ontology (GO) annotation data is downloaded from the human protein reference database (HPRD, http://www.hprd.org/) 25 to calculate the circRNA functional annotation semantic similarity. There are 19701 gene ontology data in the initial dataset. Based on circRNA-disease associations' network, 132 circRNAs related GO terms are extracted, which can be utilized to match circRNA-related gene ontology data. In this study, an information content 26 method is adopted to calculate the annotation semantic similarity of circRNA. Thus, we denote the functional annotation semantic similarity of circRNA network as *Fun_CS* and it can be calculated as follows:


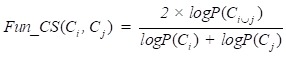
(1)

where *C_i_* and *C_j_* represent the GO terms which are related to the target genes of circRNA *C_i_* and *C_j_*, *P*(*C_i_*) and *P*(*C_j_*) denote the ratio between *C_i_* and *C_j_* target genes related GO terms and the whole GO terms, respectively. 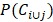 describes the proportion between the annotated GO terms on circRNA *C_i_* and *C_j_* and the whole GO terms.

#### CircRNA expression profile similarity

Expression profile data is extracted from the online database exoRBase (http://www.exorbase.org/)^27^, where has collected human circRNA and lncRNA related expression profile data. In this study, we replace the circRNA ID in circBase with the ID in exoRbase manually. Then, “Normal_circRNA_RPM” data, including the names and locations of specific chromosomes and the expression profile of circRNAs at 32 sites in normal human body, is downloaded to calculate the circRNA expression profile similarity which is denoted as *ES*. Furthermore, the Pearson correlation coefficient is adopted to measure the relevance between two circRNAs. The greater correlation score they obtain, the more similar two circRNAs are. Considering that the expression profile of two circRNA *C_i_* and *C_j_* can be expressed as 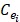 and 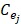. Thus, the coefficient score can be calculated as follows:


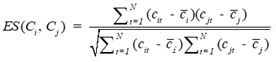
(2)

where *N* is the number of the circRNA expression profile value.

#### Fusing multiple circRNA similarity

Based on the previous calculations, three circRNA similarity networks have been constructed, including the circRNA sequence similarity network, the circRNA functional annotation semantic similarity network and the circRNA expression profile similarity network. There will be noise information in the integrated network, if only the linear method is used to integrate multiple data. Thus, a similarity network fusion 28 (SNF) algorithm is adopted to combine multiple biological data resources, which can make each network merge better and keep the most informative information.

In this study, 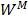 is described as the weighted matrix of each related circRNA similarity matrix and *M* is set to 3 based on three circRNA similarity networks, where 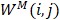 is equal to the corresponding circRNA *i* and *j* similarity score. Given that 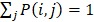 and we need to avert the scale of self-similarity in the diagonal entries. Thus, a better normalization is defined as follows:


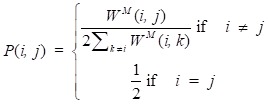
(3)

What's more, a local kernel similarity matrix of each corresponding normalized matrix can be calculated as follows:


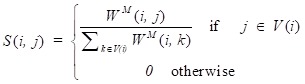
(4)

where *V*(*i*) is used to describe the *K* nearest neighbors of circRNA *i* in the integrated circRNA similarity matrix *W^M^*. Based on the above operation, the lower value neighbors of circRNA *i* are picked out and the neighbors that owe high values can be preserved, which can be illustrated as an assumption that the local similarities are more reliable than remote ones. Considering that matrix *P* takes the whole information of the various circRNA similarity information into account, while matrix *S* only carries *K* nearest neighbors' information of the network. To obtain the final circRNA similarity network, we apply the following equation to fuse multiple similarities:


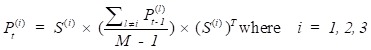
(5)

where 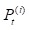 denotes the results of *t* iterations of the *ith* circRNA similarity network and the 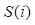 is the KNN (local) similarity matrix of the *ith* similarity matrix *P*. *M* is the number of the multiple different circRNA similarity matrices that need to be fused. In this study, *M* is equal to 3. When each matrix *P* is stable after *t* iterations, we fuse different circRNA matrixes as the following equation:


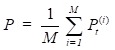
(6)

### Disease similarity

#### Disease functional similarity

To calculate disease functional similarity, disease-related genes are extracted from DisGeNet (http://www.disgenet.org/) 29 database where has collected 381,056 gene-disease associations (GDAs) between 16,666 genes and 13,172 diseases database and Online Mendelian Inheritance in Man^30^ (OMIM, https://www.ncbi.nlm.nih.gov/omim/) database. Based on the processed circRNA-disease associations dataset, 40 diseases are picked out. Thus, we use those 40 diseases to match their related genes from the above two databases. Here, a statistic method JACCARD is adopted to calculate disease functional similarity as follows:


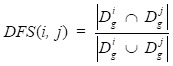
(7)

where 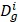 and 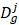 represent collections of genes associated with disease *i* and disease* j*, respectively.

#### Disease semantic similarity

There are 40 individual disease entries according to the pre-processed circRNA-disease associations. The 40 names of diseases are adopted to search their corresponding DOID manually on Disease Ontology website 31 (http://www.disease-ontology.org/). Then we use a R package called DOSE 32 to calculate the disease semantic similarity based on their relevant DOID. Thus, *DSS* is used to describe the disease semantic similarity matrix. Finally, we adopt the following equation to integrate disease functional and semantic similarities:


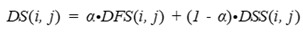
(8)

where *α* is a weighted coefficient which is used to adjust the proportion of disease functional similarity and disease semantic similarity in the final disease similarity network and the range of *α* is between 0 and 1. In this study, *α* is set to 0.5, which means that those two disease similarities are treated equally.

#### GBDTCDA

**Figure 1:**
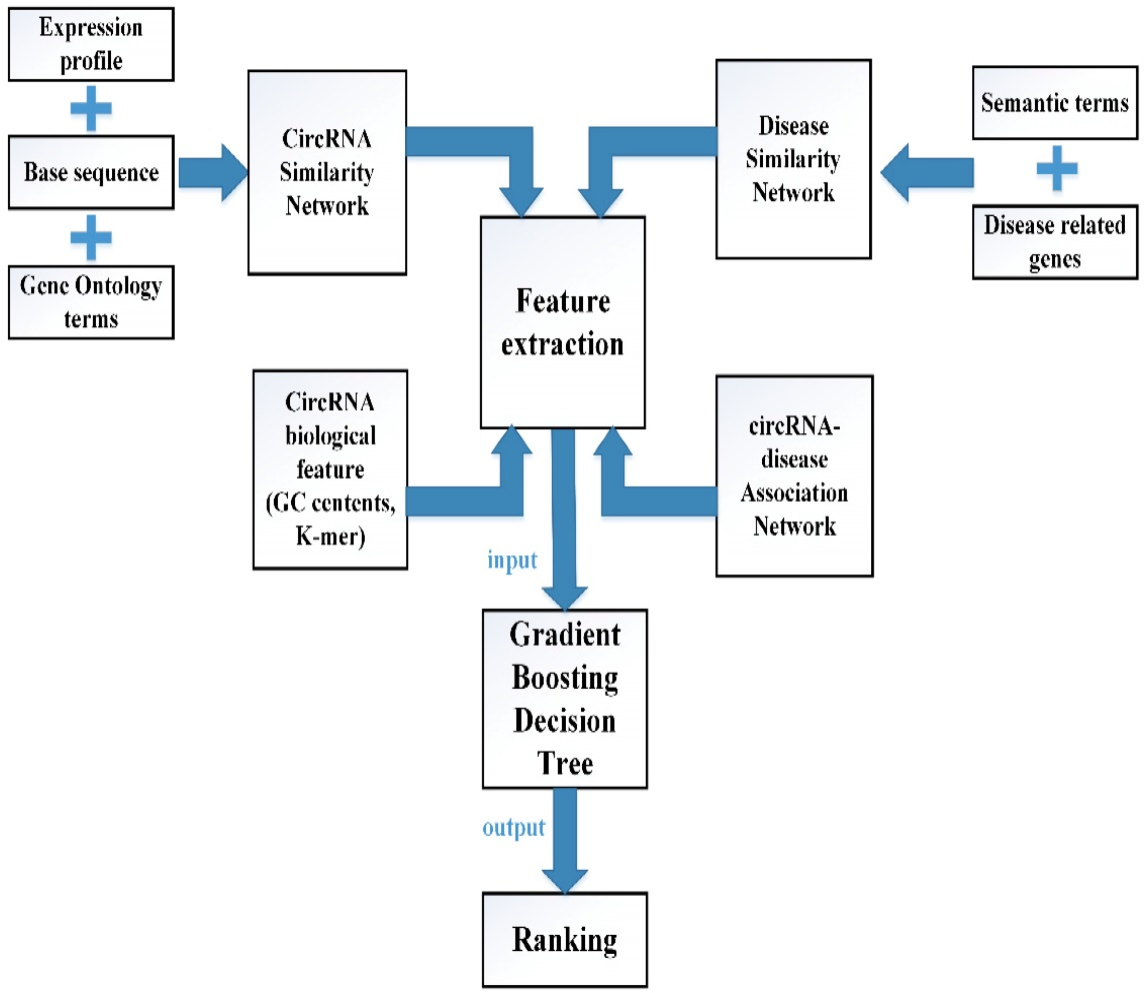
The flowchart of computational model GBDTCDA.

In this study, multiple biological data are adopted to engineer the feature vector of each circRNA-disease pair. CircRNA sequence data, circRNA expression profile and circRNA related GO terms are adopted to build the fusion circRNA similarity matrix. Disease related genes and disease phenotypes data are used to build the combined disease similarity matrix. Furthermore, circRNA nucleic acid sequence data is considered to obtain more biological information. The above data is adopted to calculate the statistic feature, graph theory feature and complex biological feature. Then, the principle component analysis (PCA) algorithm is used to extract the more essential features to reduce the noise of feature vectors. Finally, all the processed features are input into the Gradient Boosting Decision Tree machine to predict the potential circRNA-disease associations. The flowchart of our method is shown in Fig 1.

#### Engineering the feature vector

There are four different kinds of features extracted from circRNA related data which includes the integrated circRNA similarity network and circRNA nucleic acid sequence data, disease related data which contains the integrated disease similarity network and disease-circRNA associations, respectively. Some of these features are constructed in a way that we refer to previous work 33.

To extract the first type of feature for circRNA *c*(*i*) or disease *d*(*j*), some statistics information of the circRNA similarity matrix* P*, the disease similarity *DS* and the circRNA-disease associations matrix *A* are taken into our consideration. Based on the matrix *A*, *F_1.num.nei_* can be described that the number of *c*(*i*)/*d*(*j*)'s neighbors can be obtained by calculating the sum of the *ith*/*jth* column/row in the matrix *A*. Then, *F_1.sim.ave_* can be presented that the average similarity score of circRNA *c*(*i*) and disease *d*(*j*) can be calculated based on the matrix *P* and the matrix *DS*. What's more, we also take the distribution features of *c*(*i*) and *d*(*j*) similarity scores into account, which is denoted as *F_1.dis.num_*. The similarity scores [0, 1] can be divided into different distribution intervals, and then we can calculate the distribution number of similarity scores of *c*(*i*) and *d*(*j*) in each distribution interval.

To extract the second type of feature for circRNA *c*(*i*) or disease *d*(*j*), the information of graph theory of the circRNA similarity matrix *P*, the disease similarity *DS* are considered. Each similarity score of matrix *P* and matrix *DS* is used to calculate the mean similarity score. Thus, the circRNA similarity matrix *P* and disease similarity matrix *DS* can be converted into unweighted graph, when the weight of edge exceeds the average value. Then, we can use the reconstructing unweighted graph to obtain the neighbor's number of *c*(*i*) and *d*(*j*) which can be denoted as *F_2.num.nei_*. Based on the similarity matrix *P* and *DS*, we extract the top 10 similarity scores of *c*(*i*) and *d*(*j*), which can be denoted as *F_2.K.sim_*. Given the first type of circRNA and disease feature, we can calculate the average of first type feature by using the top 10 neighbors, which is described as *F_2.ave.feat1_*. Furthermore, we can also obtain the average of the first type features among the top 10 neighbors weighted by their corresponding similarity values, which are illustrated as *F_2.W.ave.feat1_*. In order to get more information of the unweighted graph, the betweenness centrality, closeness centrality and eigenvector centrality of each node in the matrix *P* and *DS* are calculated, which can be denoted as *F_2.bc_*, *F_2.cc_* and *F_2.ec_*, respectively.

In order to construct the third feature of circRNA *c*(*i*), the nucleotide sequence of *c*(*i*) is adopted to calculate the biological feature. The content of GC base in nucleic acid sequence can be regarded as an important indicator of biological characteristics, which is described as *F_3.GC.Cont_*. Then, the sequence assembly K-mer algorithm is used to count the number of matching base combination patterns, which can be denoted as *F_3.Base.K-mer_*. In this study, *K* is set as 2, 3 and 4, respectively.

For constructing the fourth feature of each circRNA-disease(*c*(*i*), *d*(*j*)) pair from the circRNA-disease associations matrix *A*. The singular value decomposition (SVD) algorithm is adopted to obtain the latent vector of *c*(*i*) and *d*(*j*), which is denoted as *F_4.svd_*. What's more, we also calculate the number of *c*(*i*)'s neighbors and the number of *d*(*j*)'s neighbors, which are described as *F_4.c.d.num_*. and *F_4.d.c.num_*. In addition, the betweenness centrality, closeness centrality and eigenvector centrality of each *c*(*i*) and *d*(*j*) pair, which can be depicted as *F_4.c.d.bc_*, *F_4.c.d.cc_* and *F_4.c.d.ec_*, respectively.

After all the information of circRNA similarity network, disease similarity network and circRNA-disease association network are extracted to construct the feature vector of each circRNA-disease pair, which are shown in supplementary Table S1. Four types of characteristics are merged into a feature vector of each circRNA-disease association as follows:


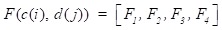
(9)

where *F_1_* is the category 1 characteristic which is the statistics information, *F_2_*is the category 2 characteristic which is the theory information, *F_3_*is the category 3 characteristic extracted from the circRNA related representational biological indicators, *F_4_*is the category 4 characteristic extracted from the cicrcRNA-disease associations network.

#### Gradient Boosting Decision Tree (GBDT) Regression

Gradient boosting^22^ is an ensemble machine learning model which combines weak 'learners' into a strong single learner in an iteration fashion. In this study, we adopt a regression tree model to train a training set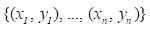 of known values of *x* and corresponding values of *y*. The objective is to find an approximation
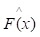
of function *F*(*x*), which minimizes the expected value of a given loss function *F_L_*(*y*, *F*(*x*)). The definition of the approximation functions 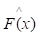
as follows:


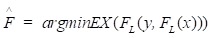
(10)

where *y* is a real value. While the gradient boosting decision tree model makes an assumption that a real-valued *y* and seeks an approximation 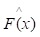
in the form of a weighted sum of functions *h_i_*(*x*) some class *H*, which can be called weak learners as follows:


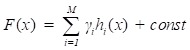
(11)

In accordance with the empirical risk minimization principle, the method tries to find an approximation 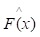 that minimizes the average value of the loss function on the training test, i.e., minimizes the empirical risk. It does so by starting with a model, consisting of a constant function *F_0_*(*x*), and incrementally expanding it using a greedy fashion:


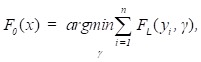
(12)


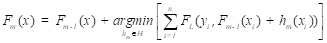
(13)

where 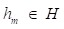 is a weak learner function.

Unfortunately, choosing the best function *h* at each step for arbitrary loss function *F_L_* is a computationally infeasible optimization problem generally. Thus, a simplified version is adopted to solve this problem.

This main thought is to apply a steepest descent step to solve this minimization problem. If we considered the continuous case, i.e. where *H* is the set of arbitrary differentiable function on *R*, we would update the model based on the following formulas:


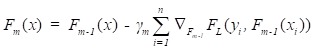
(14)


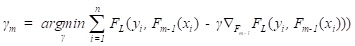
(15)

where the derivatives are taken with respect to the functions *F_i_* for *i∈*{1,..,m}^34^. In the discrete case however, i.e. when the set *H* is finite, we choose the candidate function *h* which is closest to the gradient of *F_L_* for which the coefficient *γ* may then be calculated with the aid of line search on the above equations. Note that this approach is heuristic and therefore doesn't yield an exact solution to the given problem, but rather an approximation.

#### Performance Metrics

In this study, two main evaluation metrics are adopted to estimate the performance of our computational method, such as AUC value and *F-measure*. The AUC value is the area of (receiver operating characteristic) ROC curve, which is comprised of true positive rate (*TPR*) and false positive rate (*FPR*). The following equations are adopted to calculate the *TPR* and *FPR*:


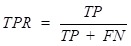
(16)


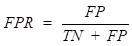
(17)

where *TP* are the known circRNA-disease associations, which are distinguished correctly, and *FN* are the unknown circRNA-disease associations, which are identified incorrectly. What's more *TN* are the unknown circRNA-disease associations, which are identified correctly. Finally, *FP* are the known circRNA-disease associations, which identified incorrectly. In addition, in order to further describe the performance of GBDTCDA, *F-measure* is also adopted to integrate* precision* and* recall*, which is a comprehensive evaluation method. *Precision* can be described as the number of true positive samples (known circRNA-disease associations) in a predicted positive sample and *recall* can be depicted as the number of positive examples in the sample predicted correctly. *F-measure* can better evaluate the performance of the model, which is calculated as follows:


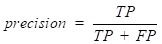
(18)


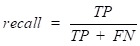
(19)


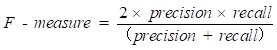
(20)

## Results

### Leave one out cross validation (LOOCV)

**Figure 2:**
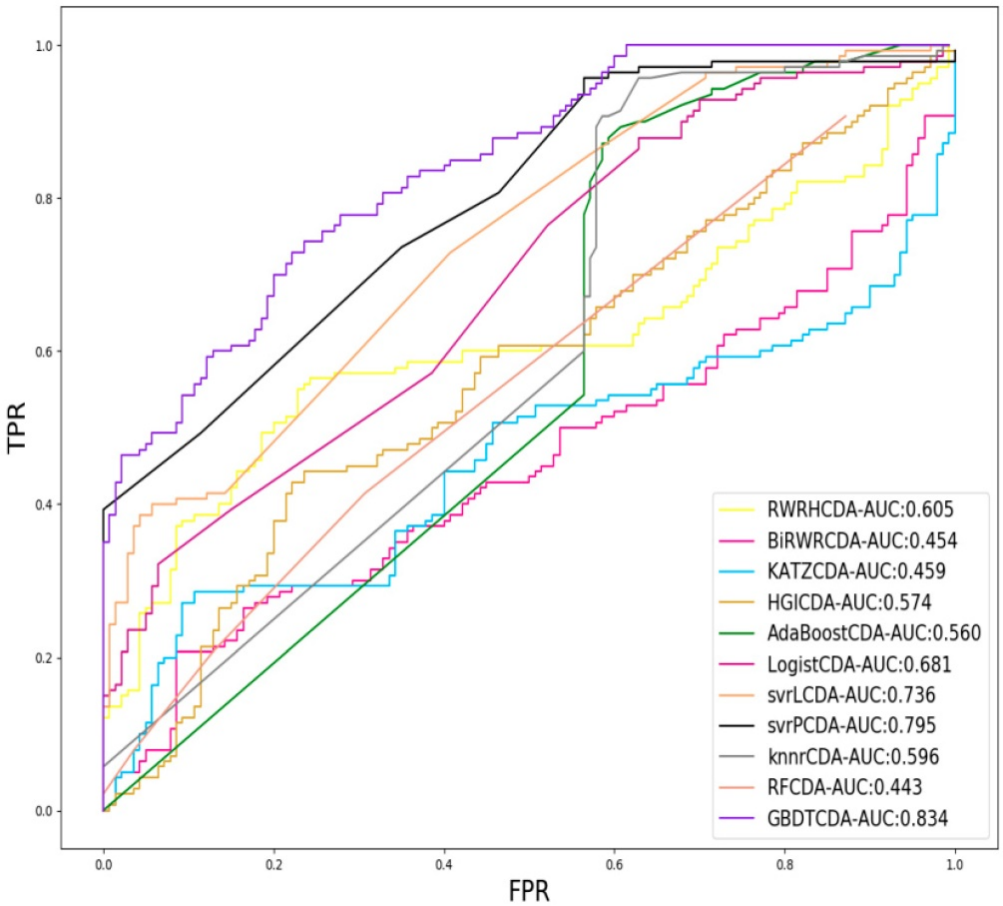
Comparison of the AUC value different methods.

For each given definite disease *i*, there are some circRNAs having associations with the disease *i*. In this study, we pick up all the known circRNA-disease associations as the positive samples and select the same number of positive samples as the negative samples in unknown relationships randomly, which adopt LOOCV to measure the performance of GBDTCDA. During each training session, one circRNA-disease association is left out as the test data. After the score of each circRNA -disease association is obtained and those scores are sorted in descending order. Each score in descending order is regarded as threshold. Along with the changing threshold, we can calculate its corresponding *TPR* and *FPR*, which can be used to draw ROC curve. For the sake of representing our proposed model can compute more accurate results than other existing methods 35-38, which is represented in Fig. 2. In this study, some state-of-the-art methods have been adopted to compare with our computational methods, which include the label propagation algorithm (random walk restart in the heterogeneous network, RWRH and bi-random walk), information flow algorithm (KATZ) and network topology algorithm (Heterogeneous graph inference, HGI). In addition, for obtaining more comprehensive evaluation of our methods, we also use some machine learning algorithm to evaluate the performance of GBDTCDA, which are consisted of ensemble learning algorithm (adapt boost, Adaboost and random forest, RF), regression algorithm (logistic regression), generalized portrait algorithm (liner and poly kernel) and neighbor learning algorithm (*k*-nearest neighbors, KNN).

**Figure 3:**
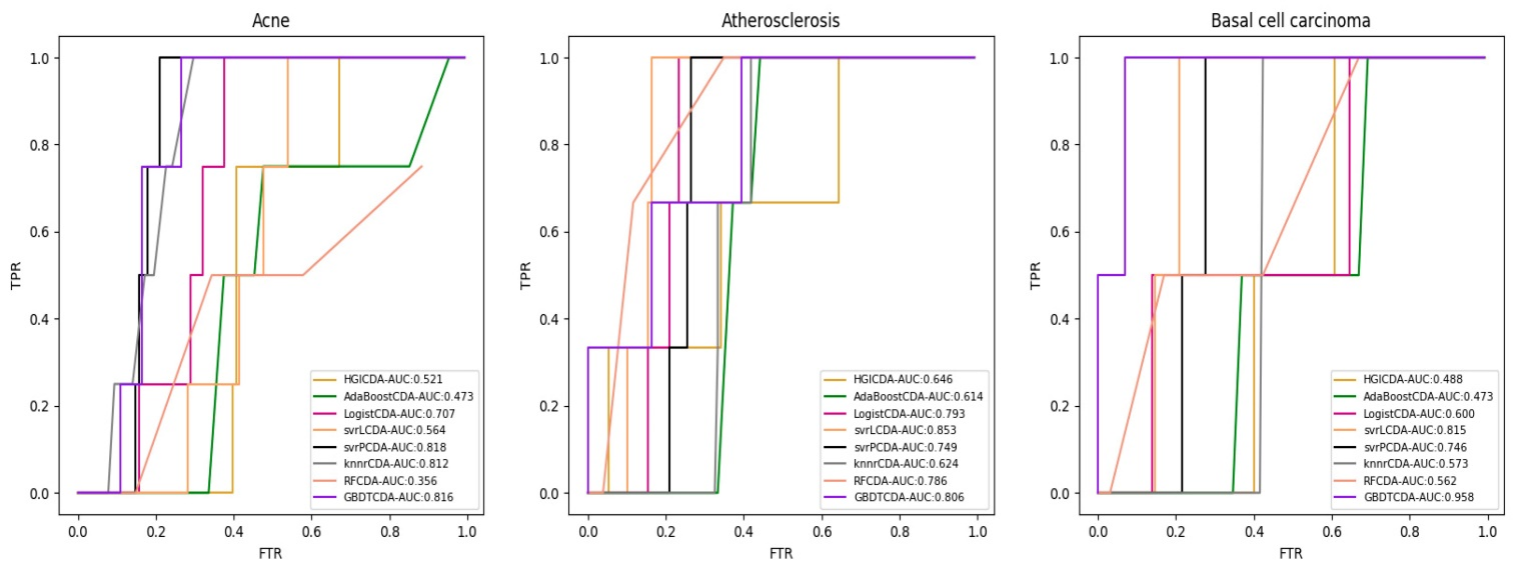
AUC value of Acne, Atherosclerosis and Basal cell carcinoma compared with other methods.

**Figure 5:**
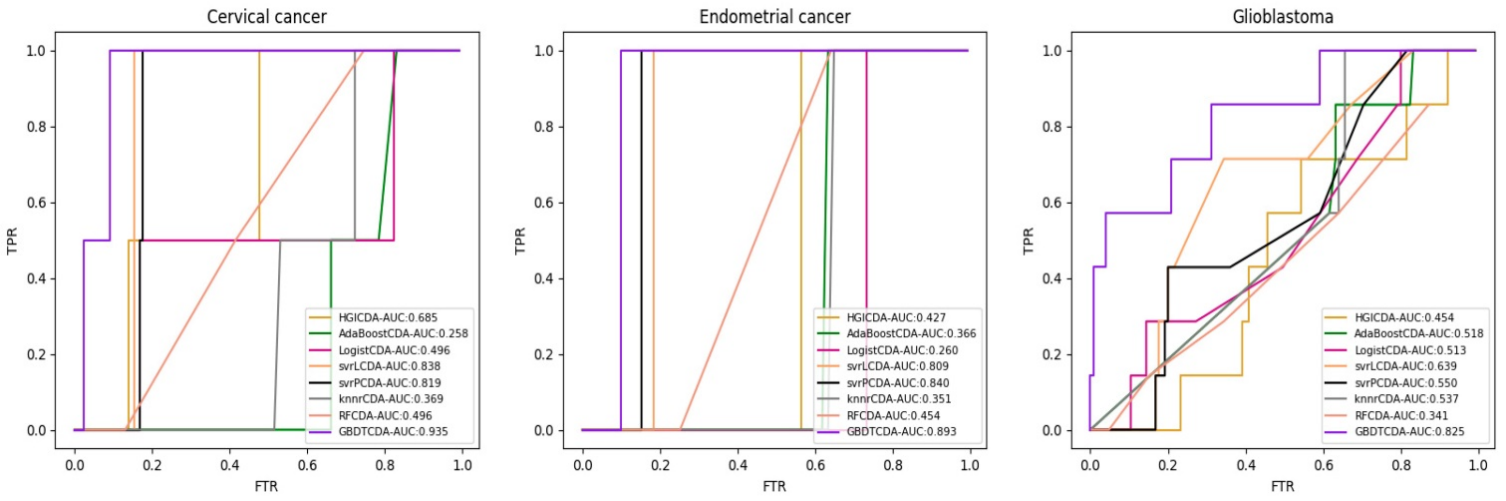
AUC value of Cervical cancer, Endometrial cancer and Glioblastoma compared with other methods.

**Figure 6:**
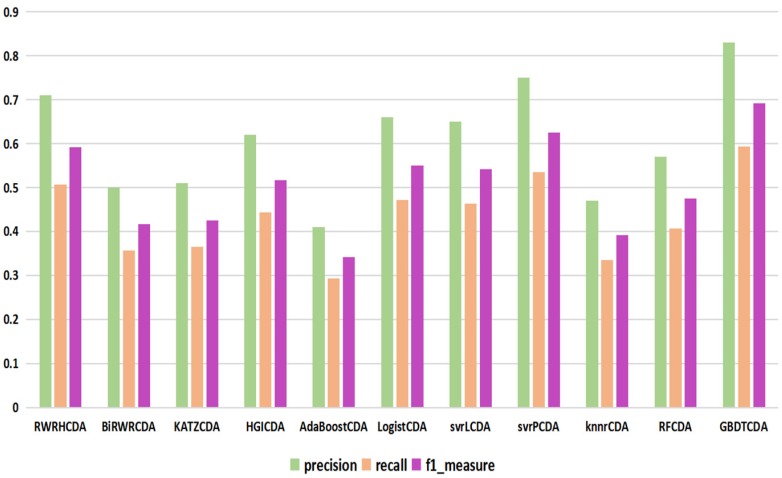
comparison of the precision, recall and f1_measure with different methods.

**Figure 7:**
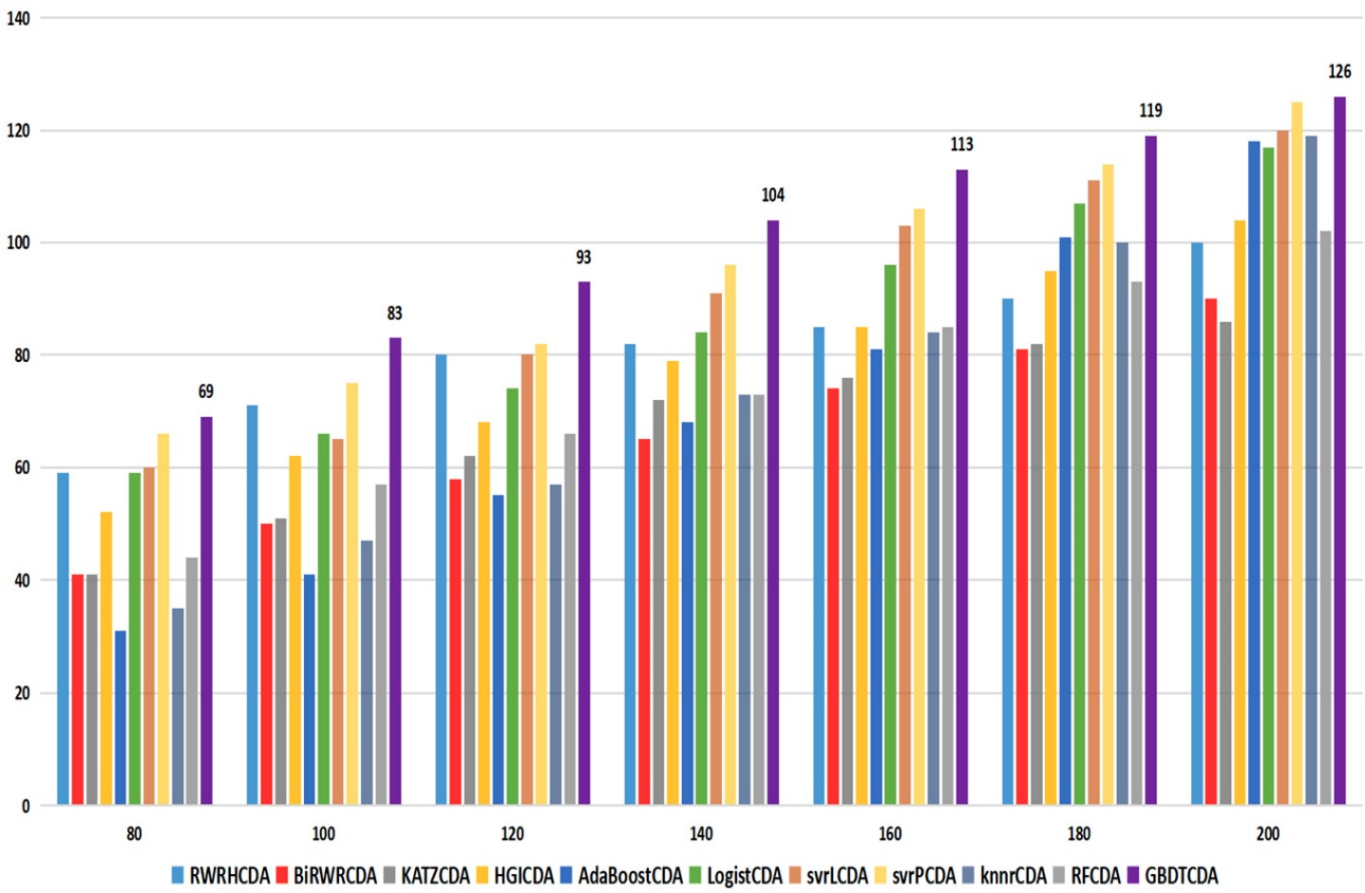
comparison of the top k ranks with different methods.

For some common disease, such as breast cancer, Colorectal cancer, Non-small cell lung cancer, Glioblastoma and other five diseases are implemented separate LOOCV experiments, respectively, which are shown in Fig. 3 to Fig. 5. In almost occasion our proposed method can obtain much better performance than other network-based algorithms and machine learning methods. For obtaining a comprehensive explanation, *F-measure* is also adopted to evaluate the performance of methods which is represented in Fig. 6. *F-measure* value of our proposed method is 0.691, which much better than other methods. What's more, for the predicting scores in top *k* (*k*∈[80, 200]) circRNA-disease associations, the number of correct circRNA-disease associations predicted by our proposed method is greater than other methods. The result is shown in Fig. 7.

### Parameter analysis

**Figure 8:**
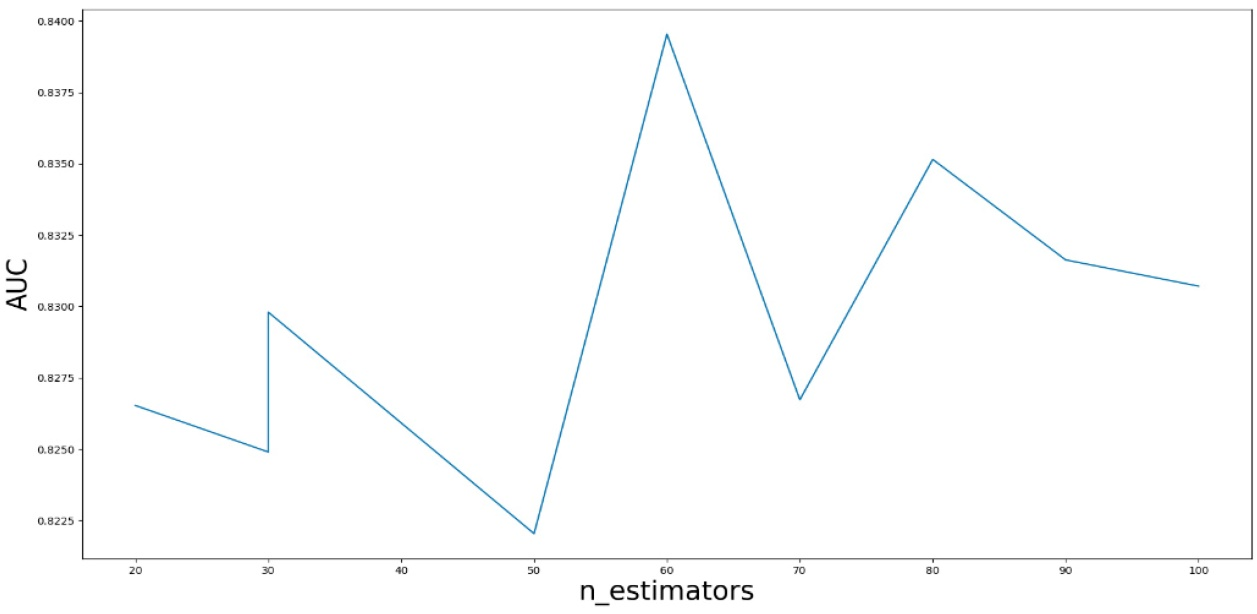
AUC value based on the different parameter n_estimators.

**Figure 9:**
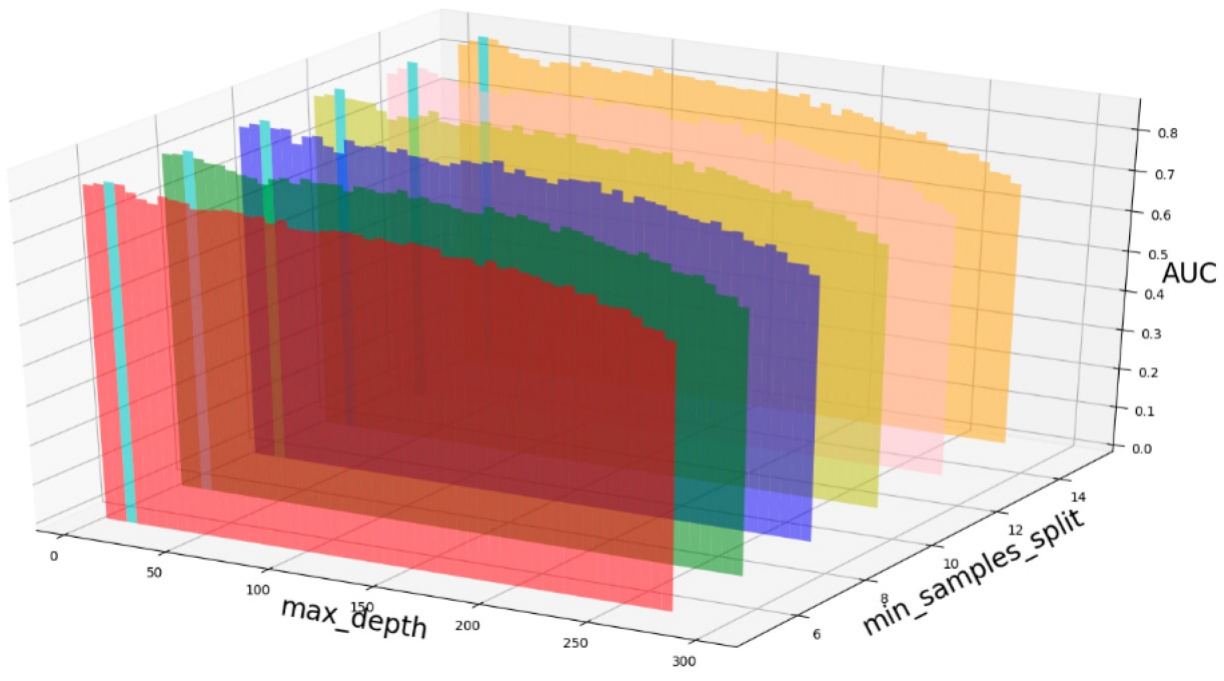
AUC value based on different max_depth and min_samples_split.

**Figure 10:**
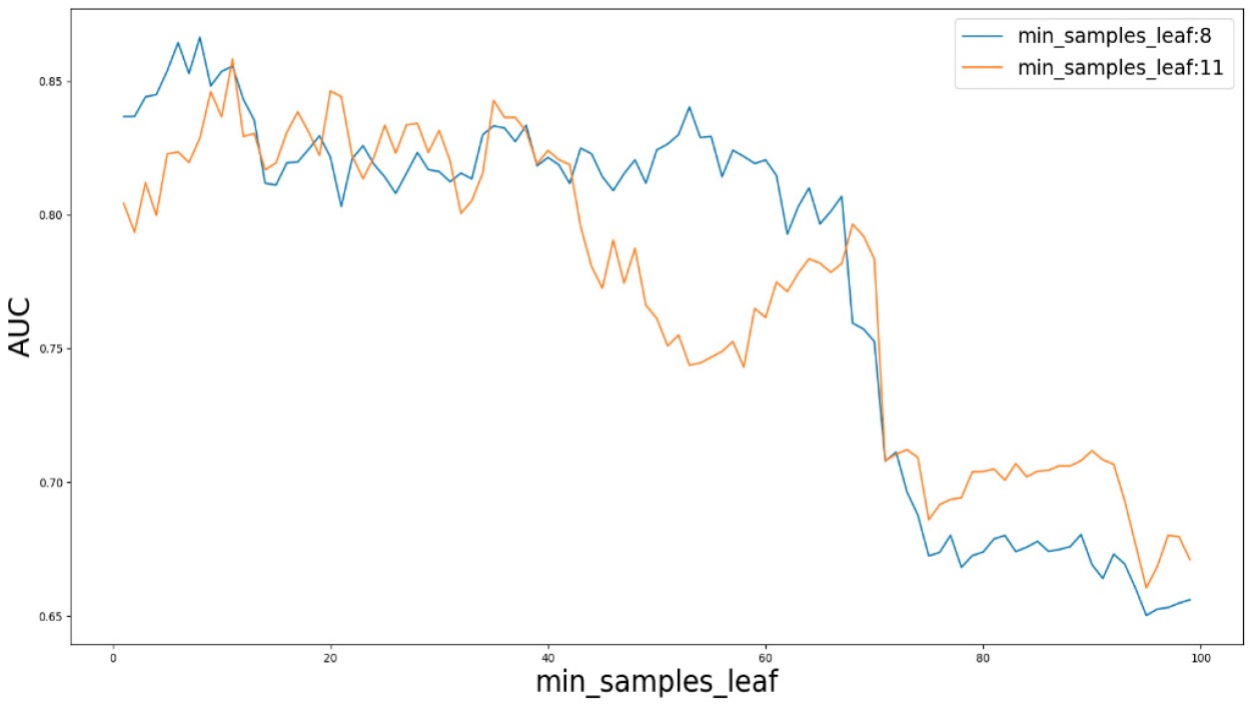
AUC value based on different parameter min_samples_leaf.

**Figure 11:**
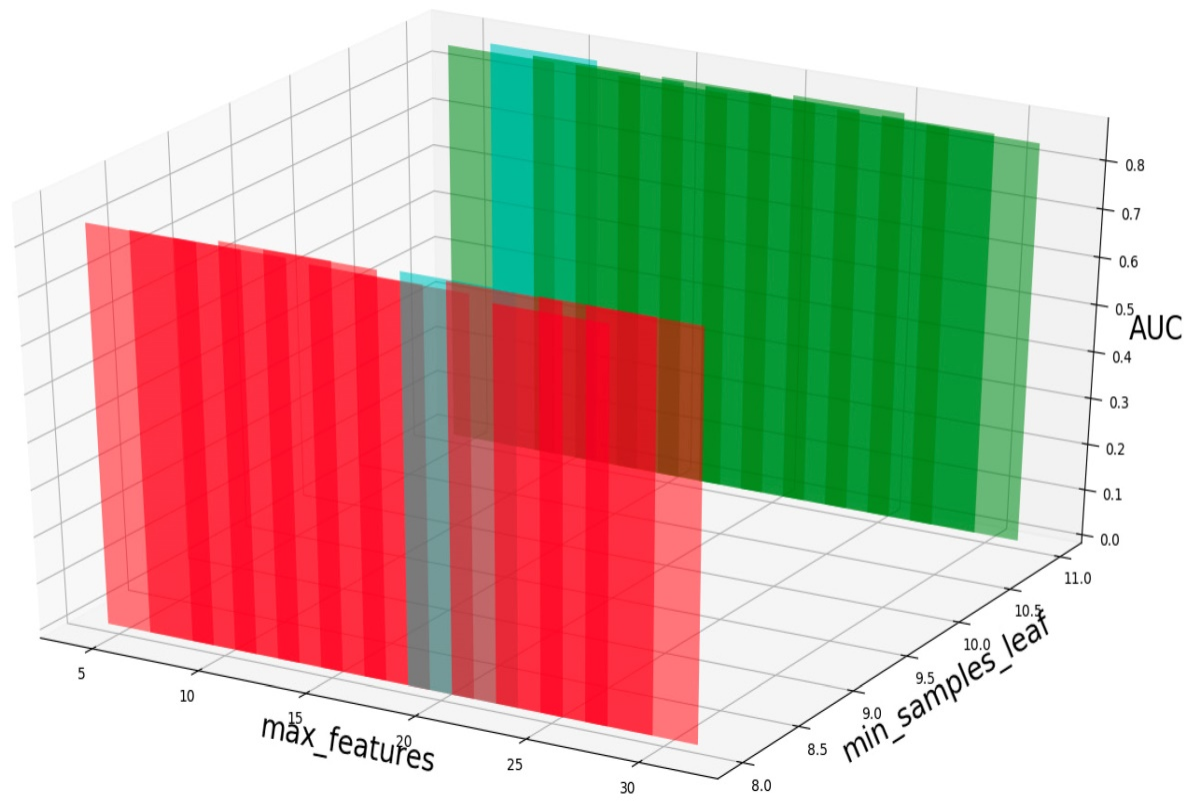
AUC value based on different min_samples_leaf and max_features.

**Figure 12:**
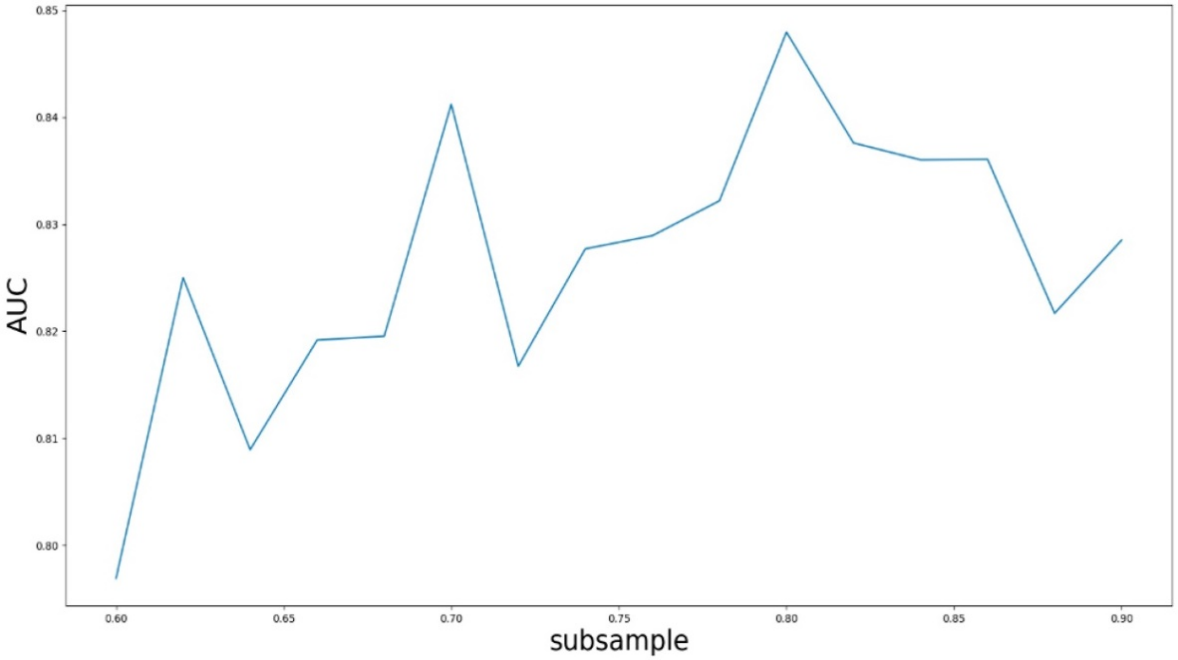
AUC value based on the different parameter subsample.

Based on our experiments, some better parameters are chosen to be set up in our computational model. There are some parameters that need to be adjusted necessarily for GBDT. Firstly, the parameter n_estimators which controls the number of trees of fit sequentially and is set up from 20 to 100 and fix parameter learning_rate as 0.1. The AUC values of different n_estimators based on the fixed learning_rate is represented in Fig. 8. When the parameter n_estimators is set as 60, the AUC value can obtain a better result. Secondly, n_estimators is fixed as 60. The parameters max_depth and min_samples_split which control the maximum depth of each decision tree and the minimum sample number for internal node repartition, respectively and they are set values from 2 to 20 and from 10 to 300, respectively. The results of the parameterization are shown in Fig. 9. Based on the results, max_depth is set as 9 and min_samples_split is set as 24. Thirdly, the parameter min_samples_leaf which control the leaf node minimum sample number is set different step sizes for adjustment, which is described in Fig 10. In according with the results, we can find that min_samples_leaf is set as 8 and 11, the maximum AUC values are obtained. Next, parameter min_samlples_leaf and max_features are adjusted together, which are presented in Fig. 11. In the light of the results, the values of min_samples_leaf and max_features are determined to be 11 and 9, respectively. Finally, the parameter subsample is set up from 0.6 to 0.9, whose results are shown in Fig. 12.

### Case study

In order to further validate the capability of predicting potential circRNA-disease associations, some case studies are made to illustrate the performance of our proposed method. The predicting results are proofed by other two circRNA-disease associations databases which are circ2Disease 39 and circRNADisease 40, respectively. In this study, one common disease is adopted to make case studies. Breast cancer 41 is one of the deadly cancers worldwide now, which also becomes a public health issue for people all over the world. Based on previous studies, some factors can increase the risk of breast cancer, such as the age of first birth 42, frequency of regular exercise and some body indices 43, diet styles 44 and environmental factors^45^. While more and more evidences illustrate that circRNAs also can be a biomarker of breast cancer, which is represented in Table 2. Based on our proposed method, the results of those predicting circRNA-disease associations are validated by database circ2Disease (C_1_) and circRNADisease (C_2_).

## Conclusion

With the rapid development of RNA high-throughput technologies, increasing number of diseases related circRNAs are discovered. Therefore, people pay more attention to revealing the intricate relationships between them. While using the traditional biological technologies are expensive and time-consuming. In this study, we propose a new computational method to predict the potential circRNA-disease associations, which is called the GBDTCDA, a machine learning driven method. what's more, gradient boosting model is first used to predict circRNA-disease associations and the LOOCV and *F-measure* evaluation measurements are adopted to illustrate the performance of our proposed method. Compared with other state-of-the-art computational methods, such as network-based methods, propagation methods and machine learning methods, GBDTCDA can get better results than those methods. In order to further describe the performance of GBDTCDA, the case studies of breast cancer are made. Thus, we believe that our proposed method GBDTCDA is a powerful tool to predict potential circRNA-disease associations.

For obtaining the better performance of our proposed computational mothed, some following significant factors cannot be ignored. Firstly, the characteristics, such as statistics information, graph theory information, circRNA sequence information and circRNA-disease latent features are taken into our consideration as comprehensive as possible, which can make the feature of each circRNA-disease pair more allover and promote the gradient boosting machine to be trained well based on those features. Secondly, in order to make our features get more reliable biological significance, multiple biological data, such as circRNA related GO terms, expression profile data and sequence data are adopted to construct the circRNA similarity network. In addition, a multiple data integration algorithm SNF is used to integrate different networks, which makes the integrated network more robust and reliable. Furthermore, an ensemble machine learning method called gradient boosting decision tree is adopted to train our inputting data.

Although our proposed method can obtain the better results compared with other methods, there still are some limits existing in our computational method. On the one hand, many parameters of gradient boosting machine need to be adjusted. In this study, the parameter adjustment is only carried out by some experiments. For our future works, some algorithms might be used to adjust those parameters. On the other hand, more categories of biological data could be taken into account, which can make our computational methods own more biological sense.

## Supplementary Material

Supplementary table S1.

## Figures and Tables

**Figure 4:**
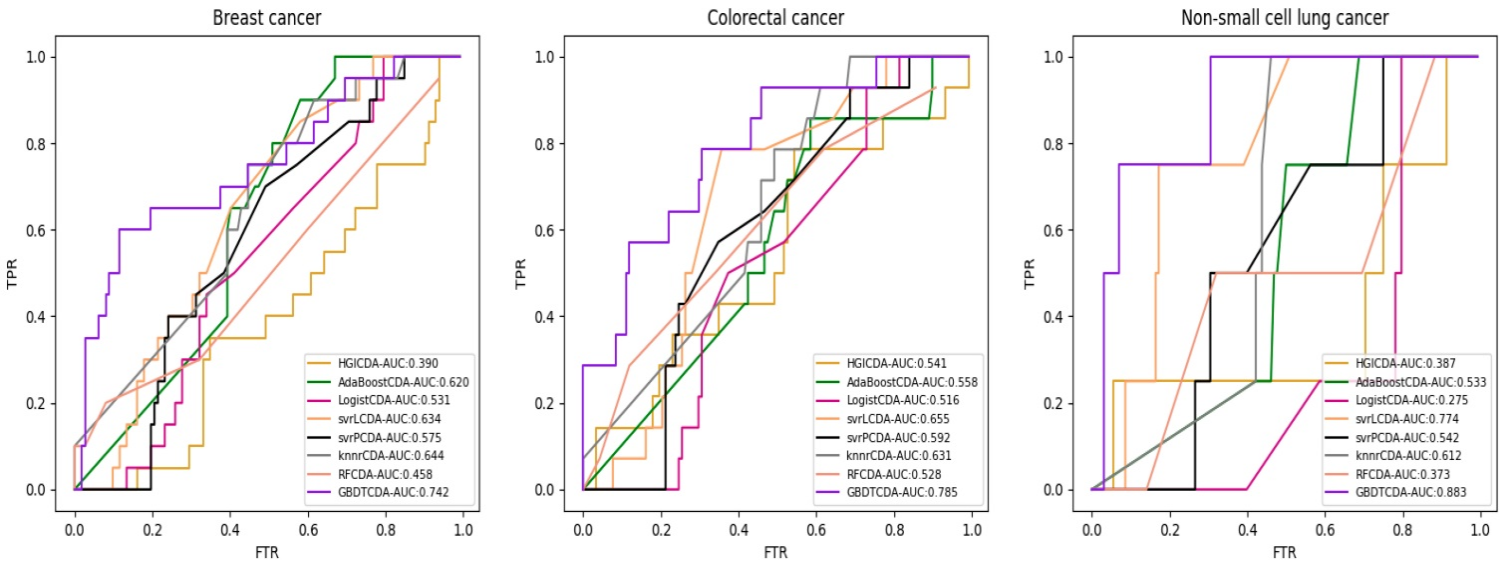
AUC value of Breast cancer, Colorectal cancer and Non-small lung cancer compared with other methods.

**Table 1:** The number of experimental data in this experiment.

Experimental data	Number
The number of circRNA-disease associations	140
The number of circRNAs	132
The number of diseases	40

**Table 2:** The top 10 breast cancer related candidates circRNAs.

Rank	circRNA name/id	Evidences	Rank	circRNA name/id	Evidences
1	hsa_circRNA_103454/hsa_circ_0067103	unconfirmed	6	hsa_circ_0007534	PMID:29593432
2	hsa_circ_0006411	unconfirmed	7	hsa_circ_0001785	C_2_
3	hsa_circ_103110/hsa_circ_0004771	C_1_, C_2_	8	hsa_circ_0001721	C_1_
4	circMYO9B/hsa_circ_0000907	PMID 29702064	9	circAmotl1/hsa_circ_0004214	C_1_, C_2_
5	circRNA_100984/hsa_circ_0002019	unconfirmed	10	hsa_circ_100219/hsa_circ_0004619	C_1_, C_2_
