# Supplementary material for: GBDTCDA: Predicting circRNA-disease Associations Based on Gradient Boosting Decision Tree with Multiple Biological Data Fusion
Source: Int J Biol Sci. 2019 Nov 8;15(13):2911–24. doi: 10.7150/ijbs.33806 (PMC6909967; doi:10.7150/ijbs.33806)
Supplement: Supplementary file 1 — Supplementary table S1. [file ijbsv15p2911s1.pdf]

Table S1. Feature vector component of each circRNA-disease pair.

First type of feature for circRNA/disease (F_1)	$\mathbf{F_{1.num.nei}}$	The number of c(i)/d(j)'s neighbors
	$\mathbf{F_{1.sim.ave}}$	The average similarity score of circRNA c(i) and disease d(j)
	$\mathbf{F_{1.dis.num}}$	The distribution number of similarity scores of c(i) and d(j) in each distribution interval
Second type of feature for circRNA/disease (F_2)	$\mathbf{F_{2.num.nei}}$	The neighbor's number of c(i) and d(j) based on the unweighted circRNA/disease similarity network
	$\mathbf{F_{2.K.sim}}$	The top 10 similarity scores of c(i) and d(j)
	$\mathbf{F_{2.ave.feat1}}$	The average first type feature of c(i) and d(j) by the top 10 neighbors
	$\mathbf{F_{2.W.ave.feat1}}$	The average of the first type features among the top 10 neighbors weighted by its corresponding similarity values
	$\mathbf{F_{2.bc}}$,$\mathbf{F_{2.cc}}$,$\mathbf{F_{2.ec}}$	Betweenness centrality, closeness centrality and eigenvector centrality of each c(i) and d(j) node
Third type of feature of circRNA (F_3)	$\mathbf{F_{3.GC.Cont}}$	The content of GC base in nucleic acid sequence
	$\mathbf{F_{3.Base.K\text{-}mer}}$	The sequence assembly K-mer algorithms is used to count the number of matching base combination patterns
Forth type of feature of circRNA-disease association pair (F_4)	$\mathbf{F_{4.svd}}$	Latent vector of c(i) and d(j) based on SVD algorithm
	$\mathbf{F_{4.c.d.num}}$	The number of circRNA c(i)'s neighbors
	$\mathbf{F_{4.d.c.num}}$	The number of disease d(j)'s neighbors
	$\mathbf{F_{4.c.d.bc}}$,$\mathbf{F_{4.c.d.cc}}$,$\mathbf{F_{4.c.d.ec}}$	The betweenness centrality, closeness centrality and eigenvector centrality of node in cicrRNA-disease associations network
